# Specific antioxidant compounds differentially modulate cytotoxic activity of doxorubicin and cisplatin: *in vitro* and *in vivo* study

**DOI:** 10.3325/cmj.2014.55.206

**Published:** 2014-06

**Authors:** Rostyslav Panchuk, Nadia Skorokhyd, Vira Chumak, Lilya Lehka, Sofya Omelyanchik, Valery Gurinovich, Andrey Moiseenok, Petra Heffeter, Walter Berger, Rostyslav Stoika

**Affiliations:** 1 Institute of Cell Biology, National Academy of Sciences of Ukraine, Lviv, Ukraine; 2 Ivan Franko Lviv National University, Lviv, Ukraine; 3 Center of Food, National Academy of Sciences of Belarus (Grodno Branch), Grodno, Belarus; 4 Institute of Cancer Research, Vienna Medical University, Vienna, Austria

## Abstract

**Aim:**

To use the antioxidant compounds (sodium selenite, selenomethionine, D-pantethine) for modulation of cytotoxic effect of doxorubicin and cisplatin toward wild type and drug-resistant mutants of several human tumor cells. Similar treatments were applied *in vivo* toward adult male Wistar rats.

**Methods:**

Human tumor cells of different lines (HCT-116, Jurkat and HL-60) with various mechanisms of drug-resistance were treated with doxorubicin or cisplatin, alone or in combination with sodium selenite, selenomethionine, or D-pantethine. Cell viability, induction of apoptosis, and production of O_2_^-^ radicals were measured. Activity of redox potential modulating enzymes was measured in the liver and blood plasma of adult male Wistar rats subjected to similar treatments.

**Results:**

All antioxidants used in physiologically harmless concentration inhibited cytotoxic action of doxorubicin toward tumor cells sensitive to chemotherapy treatment by 15%-30%, and slightly enhanced cytotoxic effect of this medicine toward drug-resistant malignant cells. At the same time, there was no significant effect of these antioxidants on cisplatin action. Such effects were accompanied by a complete inhibition of production of superoxide radicals induced by doxorubicin. The results of *in vivo* study in adult male Wistar rats were in agreement with the results of *in vitro* study of human tumor cells.

**Conclusion:**

Protective effect of specific antioxidant agents during cytotoxic action of doxorubicin was demonstrated *in vitro* in drug-sensitive human tumor cells and in adult male Wistar rats, while there was no protective effect in drug-resistant sub-lines of these tumor cells during action of doxorubicin and cisplatin.

Low selectivity of action of the chemotherapeutic agents is one of their main shortcomings, leading to serious negative side effects in cancer patients. The main reason for this phenomenon is the formation of free radicals during the action of these drugs in both normal and tumor cells. Doxorubicin and cisplatin are among the most commonly used anticancer drugs. They realize the antineoplastic activity by the intercalation into DNA structure and production of the reactive oxygen species (ROS) (1-3). However, these drugs lead to severe cardio- and nephrotoxicity, which significantly limits their use for tumor treatment (4). It was shown that side effects of doxorubicin and cisplatin are mediated by hydroxyl radicals, which are formed in the presence of iron (II) from superoxide anions whose production is induced by these drugs (3,5). Numerous studies indicate that ROS-induced apoptosis of tumor cells takes place only under supraclinical doses of anthracyclines, and ROS production is not critical for realization of their anticancer activity (3). Thus, selective blocking of ROS action by specific antioxidant agents should at least partially reduce the toxicity of doxorubicin and cisplatin toward normal cells, without significant impact on the antitumor action of these drugs. Promising candidates for such role are derivatives of the pantothenic acid, since they possess significant antioxidant effect toward the mammalian cells and are able to protect the cells against toxic effects of free radicals (6). The inorganic and organic selenium derivatives (sodium selenite and selenomethionine) belong to another group of antioxidants that demonstrated a protective effect during cisplatin chemotherapy (7,8). Similar protective effects were also observed for the pantothenic acid (9). However, it remains unknown whether these antioxidants are capable of inhibiting the production of harmful ROS (including superoxide and hydroxyl radicals) due to the action of anticancer agents, and at the same time not interfering with the anti-tumor activity of these drugs. Besides, the effect of D-pantethine, selenomethionine, and sodium selenite used in combination with the anticancer drugs toward tumor cells resistant to chemotherapy has not been studied thoroughly (10).

In this study, we aimed to develop new approaches for cancer chemotherapy that would eliminate negative side effects of the anticancer drugs caused by an excessive production of free radicals, which adversely affect normal tissues and organs in cancer patients. A chemotherapy regimen based on a combination of specific antioxidants (sodium selenite, selenomethionine, D-pantethine) and conventional anticancer drugs (doxorubicin, cisplatin), which are known to induce production of ROS, has been proposed. We studied the molecular mechanisms of antitumor activity of doxorubicin and cisplatin combined with the antioxidants toward tumor cell lines possessing different mechanisms of drug resistance. The results obtained in the *in vitro* study have been verified in experimental animals (rats).

## Materials and methods

The study was conducted at the Institute of Cancer Research Vienna, Austria, the Institute of Cell Biology, Lviv, Ukraine, and the Center of Food, National Academy of Sciences of Belarus in Grodno in 2013. Human isogenic p53-null (p53−/−), Bax-null (Bax−/−), and wild-type (p53+/+, Bax +/+) human HCT-116 colon carcinoma cells (kindly provided by Dr Bert Vogelstein), human breast adenocarcinoma cells of MCF-7 line, human T-leukemia cells of Jurkat line, human leukemia cells of HL-60 line, and its drug-resistant HL-60/vinc sub-line (overexpression of P-glycoprotein) were obtained from cell culture collection at the Vienna Medical University, Institute of Cancer Research. Cells were cultured in RPMI-1640 medium, supplemented with 10% fetal calf serum (Sigma Chemical Co., St. Louis, MO, USA), 50 µg/mL streptomycin (Sigma Chemical Co.), and 50 units/mL penicillin (Sigma Chemical Co.) in 5% CO_2_-containing humidified atmosphere at 37°C. Cells were seeded into 24-well tissue culture plates (Greiner Bio-one, Frickenhausen, Germany). Short-term (24 hours) cytotoxic effect of anticancer drugs was studied under the Evolution 300 Trino microscope (Delta Optical, Mińsk Mazowiecki, Poland) after cell staining with Trypan blue (0.1%).

To analyze cytotoxic activity of conventional anticancer drugs together with non-toxic doses of the antioxidants *in vitro*, tumor cell lines possessing various mechanisms of resistance to anticancer drugs were studied. For estimating the impact of the antioxidants on cytotoxic activity of conventional anticancer drugs, semi-lethal doses of cisplatin and doxorubicin causing death of 50% of malignant cell were used in combination with non-toxic doses of the antioxidants. The effect of antioxidants that led to a 5%-10% (statistically unreliable) decrease in cytotoxic activity of drugs was considered weak, the effect that led to a decrease of 15%-30% was considered moderate, and that which led to a decrease of 30%-50% was considered strong.

The cells were stained with DAPI (4',6-diamidino-2-phenylindole) (Sigma) for studying chromatin condensation in the MCF-7 cells treated with doxorubicin and antioxidants. 24 hours after the addition of drugs, MCF-7 cells were washed twice with 1x PBS, fixed for 15 minutes at room temperature in 4% solution of paraformaldehyde, and permeabilized for 3 minutes with 0.1% Triton X-100 in the phosphate buffer saline (PBS). After that, cells were incubated for 5 minutes with 1 µg/mL solution of DAPI (Sigma), washed twice with PBS, and cover glasses with fixed cells were placed on slides. Cytomorphological study was performed under Carl Zeiss AxioImager A1 fluorescent microscope (Carl Zeiss, Göttingen, Germany).

The content of the ROS was measured after incubation of control or drug-treated (24 hours) MCF-7 cells with fluorescent dye dihydroethidum (DHE, O_2_^-^-specific) used in 10 μM concentration for 30 minutes at 37°C. After incubation with ﬂuorochrome, cover glasses with cells were washed with PBS and placed on slides, and the intensity of fluorescence was immediately analyzed under Carl Zeiss AxioImager A1 fluorescent microscope (Carl Zeiss).

Thirty-two adult male Wistar CRL rats with 90-110 g weight were kept under standard vivarium conditions with constant access to the full feed and drinking water. Animals were divided into 4 groups of 8 rats each. Rats from experimental groups were intragastrically administered D-pantethine 400 mg/kg (group 3) and selenomethionine 200 µ/kg (group 4) for 5 days. Rats from the first (control) group were injected simultaneously with equivalent volume of 0.9% sodium chloride solution in a similar mode. Doxorubicin (5 mg/kg) was injected once i.p. to the animals from 2-4th groups in 3 days before decapitation.

All *in vivo* experiments were conducted in accordance with the international principles of the European Convention for protection of vertebrate animals under a control of the Bio-Ethics Committee of the Center of Food, National Academy of Sciences of Belarus in Grodno.

The activity of glutathione reductase and glutathione transferase was measured according to Carlberg et al (11,12). Glutathione and its redox potential in erythrocytes was determined as previously described (13). Products that react with N, N-dimethyl-p-phenylenediamine in plasma were detected as previously described (14). Analysis of free SH-groups in proteins from blood plasma was done as previously described (15).

Activity of succinate dehydrogenase and 2-oxoglutarate dehydrogenase was measured spectrophotometrically (16). Sorption reaction with Nile blue dye was used as a functional test of the biological stability of erythrocyte membranes, as previously described (17). The level of adsorption in erythrocytes was measured by using methylene blue assay (18).

Identification of coenzyme A (CoA) fractions was conducted as previously described (19,20). The content of free form of CoA (CoA-SH) and short chain acyl-CoA derivative (acetyl- CoA) in the rat liver was determined using HPLC assay on a HPLC instrument Agilent 1100/1200 (Agilent Technologies, Santa Clara, CA, USA). Homogenization of liver tissue samples was performed at 4°C using 4% HClO_4_ at a ratio of 1:6. The homogenates were centrifuged at 16 000 g for 15 minutes at 4°C and the obtained chlorine supernatants were adjusted to pH 5 with 20% NaOH. Prior to introduction into the chromatograph, the supernatant was filtered through a RC 0.45 μm, 13 mm filter (Agilent Technologies). Chromatographic column Zorbax SB-C_18_ 150x3 mm, particle size 3.5 µm (Agilent Technologies) was used for HPLC.

All experiments were performed in triplicate and repeated 3 times. For statistical analysis, standard variation data within a group were calculated together with a statistical reliability of differences between two groups of data assessed by *t*-test. The level of significance was set to 0.05.

## Results

### Differential effect of antioxidants toward cytotoxic action of doxorubicin in drug-sensitive and drug-resistant tumor cell lines

**Figure 1:**
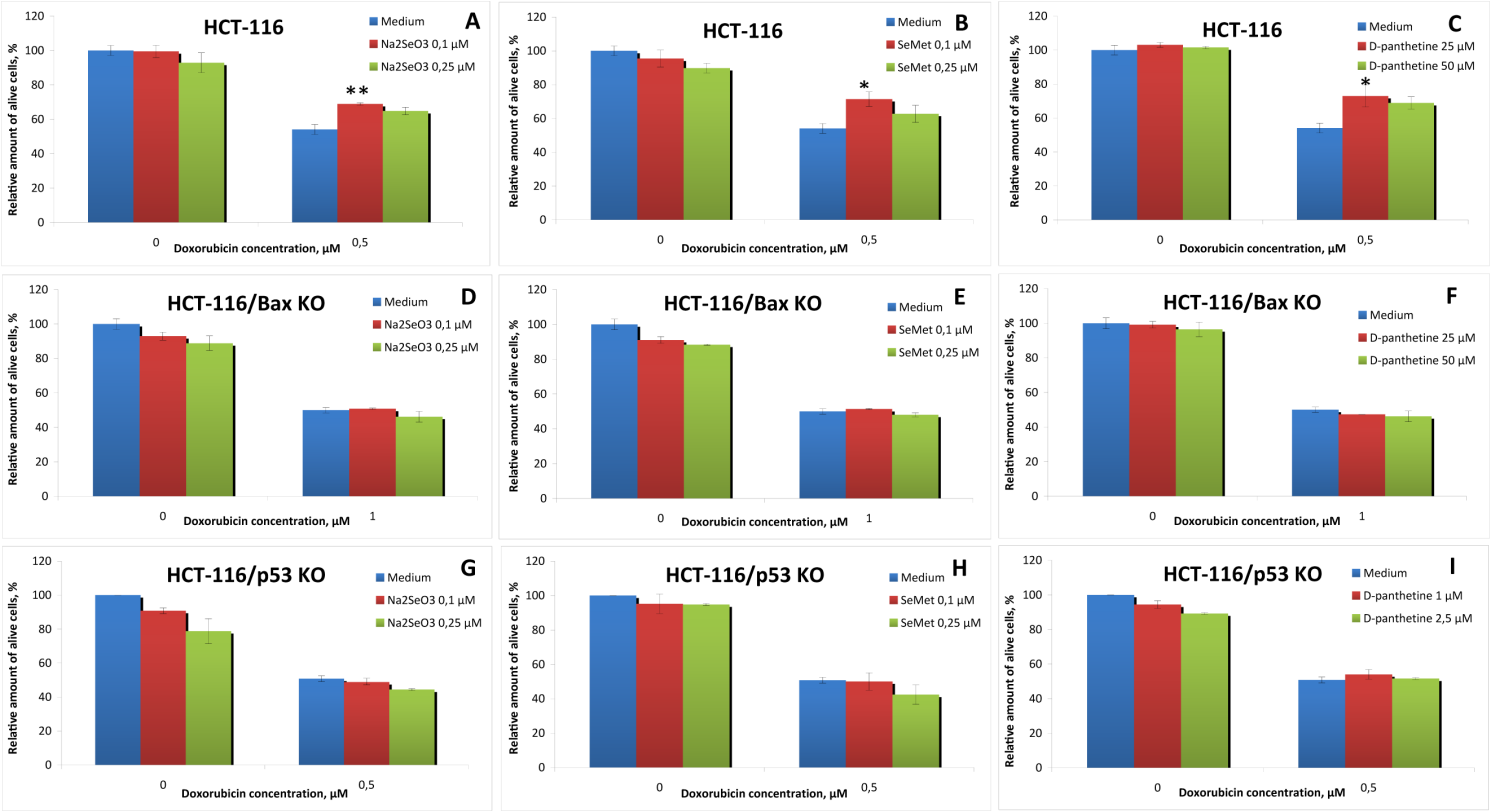
Sodium selenite, selenomethionine, and D-pantethine decrease cytotoxic action of doxorubicin toward wild-type HCT-116 cells, but have no impact on doxorubicin action toward HCT-116/Bax(−/−) and HCT-116/p53(−/−) sub-lines lacking *Bax* and *p53* genes. Medium – blank control (appropriate volume of culture medium added instead of the antioxidants). *P < 0.05 relative to the control. A – *P* = 0.0098, B – *P* = 0.0211, C – *P* = 0.0321.

Human colon carcinoma cells of HCT-116 wild-type line (with intact copies of the p53 and Bax genes) and its sub-lines HCT-116 (Bax (−/−) and HCT-116/p53 (−/−) characterized by a knockout of Bax and p53 genes, respectively, were chosen as an experimental model. Deletion of Bax gene resulted in a 2-fold increase in the resistance of these cells to doxorubicin action, whereas a deletion of the p53 gene did not affect this parameter (Figure 1). Sensitivity to sodium selenite and selenomethionine in HCT-116 wild-type cells and its gene knockout HCT-116/p53 (−/−) and HCT-116/Bax (−/−) sub-lines was almost identical. These compounds used in 0.1-0.25 µM doses were completely non-toxic toward the studied cell lines (Figure 1). All applied antioxidants demonstrated moderate cytoprotective action, decreasing by 15%-20% the cytotoxic effect of doxorubicin toward HCT-116 cells (Figure 1). On the contrary, sodium selenite and selenomethionine in the same dose (0.1 µM) did not protect HCT-116/Bax (−/−) and HCT-116/p53 (−/−) sub-lines, while in a higher and still non-toxic concentration (0.25 µM) they only slightly intensified doxorubicin action (Figure 1). Surprisingly, HCT-116/p53 (−/−) cells were hypersensitive to D-pantethine action, and thus 20 times lower concentrations of this antioxidant (1 µM and 2.5 µM) were used compared to doses applied toward wild-type cells (25 µM and 50 µM, respectively). However, even in these low doses, D-pantethine did not act cytoprotectively toward HCT-116/p53 (−/−) and HCT-116/Bax (−/−) cells, while it protected HCT-116/wt cells with the same or even higher efficiency than did the selenium derivatives. Thus, antioxidants seem to protect against toxic effect of doxorubicin only drug-sensitive tumor cells, and have no impact on cytotoxic action of doxorubicin toward tumor cells with impaired structure of *Bax* and *p53* genes.

**Figure 2:**
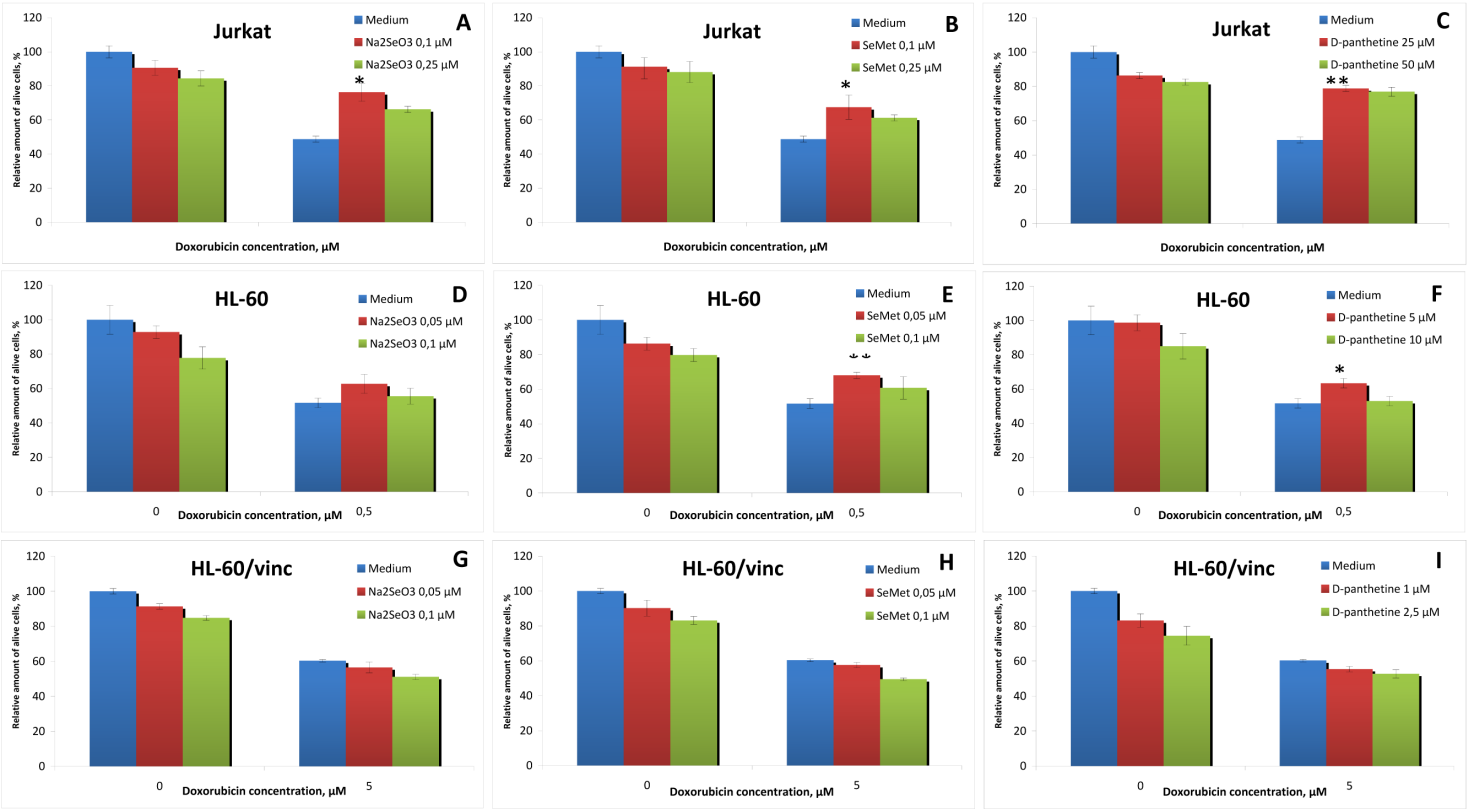
Sodium selenite, selenomethionine, and D-pantethine decrease cytotoxic action of doxorubicin toward human leukemia cells of Jurkat line and HL-60 line, but have no impact on doxorubicin action toward vincristin-resistant HL-60/vinc sub-line overexpressing P-glycoprotein. Medium – blank control (appropriate volume of culture medium added instead of antioxidants). *P < 0.05 relative to the control, **P < 0.01 relative to the control. A – *P* = 0.010, B – *P* = 0.034, C – *P* = 0.002, E – *P* = 0.004, F – *P* = 0.026.

In order to confirm the obtained results, human leukemia cells of HL-60 line and its drug-resistant HL-60/vinc sub-line characterized by an overexpression of P-glycoprotein, were used. HL-60 cells are very sensitive to doxorubicin action (LC_50_ = 0.5 µM), and sodium selenite, selenomethionine, and D-pantethine demonstrated weak protective effect against the action of this anticancer drug (Figure 2). In contrast, the cells of HL-60/vinc sub-line showed a 10-fold increase in their resistance to doxorubicin, while all applied antioxidants failed to have a protective effect in these cells (Figure 2). Human T-leukemia cells of Jurkat line were most sensitive to doxorubicin action, and they also responded distinctly on the protective action of sodium selenite, selenomethionine, and D-pantethine (Figure 2). In particular, sodium selenite in 0.05 µM dose or D-pantethine in 25 µM dose acting together with doxorubicin in its LC_50_ dose (0.5 µM) toward Jurkat T-cells significantly recovered the cell population to 78%-80% of the control level, while the effect of selenomethionine was slightly weaker. It should be noted that similar to HCT-116 cells, a slight increase (from 0.05 to 0.1 µM) in the concentration of these antioxidants decreased their cytoprotective effect (Figure 2).

### Cytotoxic action of cisplatin is not affected in tumor cells by the antioxidants

**Figure 3:**
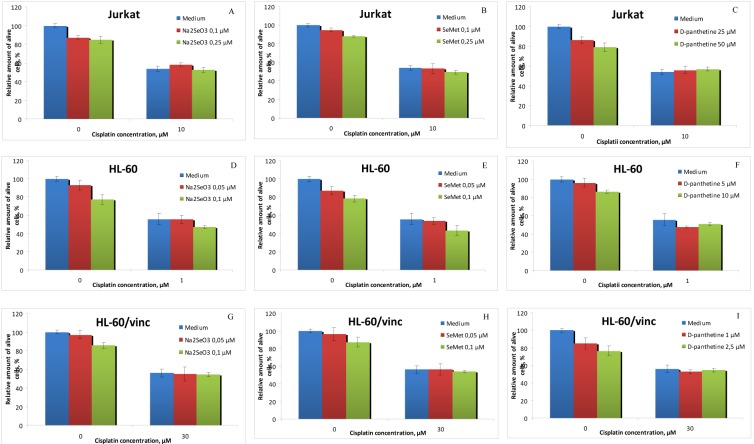
Sodium selenite, selenomethionine, and D-pantethine have no impact on viability of human leukemia cells of Jurkat line, HL-60 line, and its vincristin-resistant HL-60/vinc sub-line, overexpressing P-glycoprotein under cytotoxic action of cisplatin. Medium – blank control (appropriate volume of culture medium added instead of antioxidants).

**Figure 4:**
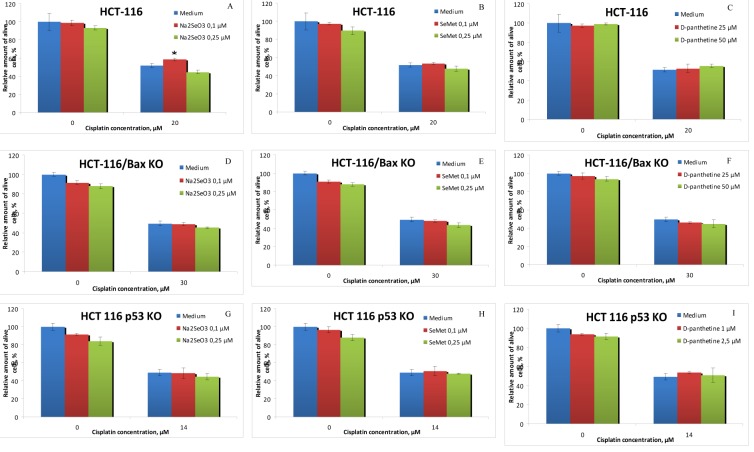
Sodium selenite, selenomethionine, and D-pantethine have no impact on viability of HCT-116 cells and their HCT-116/Bax(−/−) and HCT-116/p53(−/−) sub-lines lacking *Bax* and *p53* genes under cytotoxic action of cisplatin. Medium – blank control (appropriate volume of culture medium added instead of antioxidants). *P < 0.05 relative to the control. A – *P* = 0.035.

In contrast to doxorubicin action, there was no cytoprotective effect of the antioxidants in the case of cisplatin action. A combined treatment of Jurkat T-leukemia cells with LC_50_ dose of cisplatin (10 µM) and 0.1 µM of sodium selenite led to only a 4% decrease in cisplatin cytotoxicity, while selenomethionine and D-pantethine had no effect at all (Figure 3). The same lack of effect was observed in the case of HL-60 leukemia cells, as well as its drug-resistant HL-60/vinc sub-line (Figure 3). There was only a weak cytoprotective effect of sodium selenite of 7%, and a lack of effect of selenomethionine and D-panthenine toward cisplatin cytotoxic action in the parental HCT-116 cell line, as well as its drug-resistant Bax (−/−) and p53 (−/−) sub-lines (Figure 4). Thus, drug-resistant tumor cells compared with drug-sensitive tumor cells showed much weaker sensitivity to cisplatin action, which is similar to a decreased sensitivity to antioxidants’ action in the case of doxorubicin.

### Antioxidants inhibit ROS production and apoptosis induced by doxorubicin in tumor cells

Sodium selenite, selenomethionine, and D-pantethine only partially (15%-30%) suppressed cytotoxic activity of doxorubicin in drug-sensitive tumor cells (Figure 1,2). Since ROS production is a supplementary pathway in apoptosis induced by doxorubicin (3), it is obvious that the observed effect of antioxidants is explained by the inhibition of production of superoxide anions under the doxorubicin treatment.

**Figure 5:**
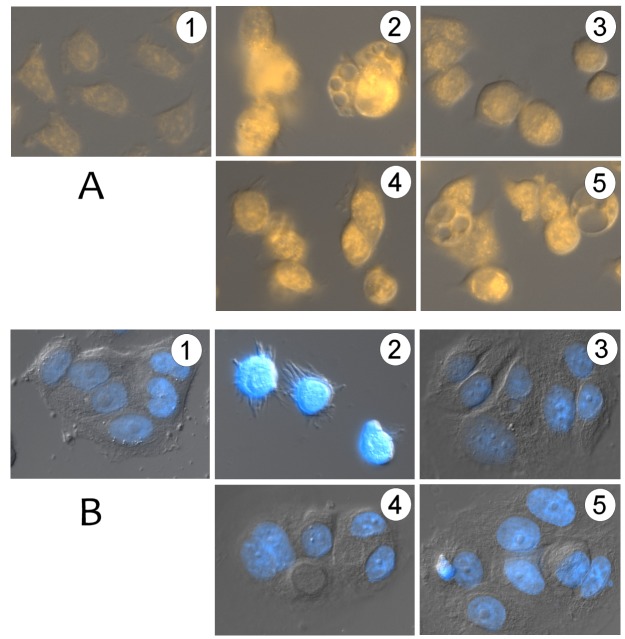
Sodium selenite, selenomethionine, and D-pantethine inhibit production of superoxide anions (**A**) and induction of apoptosis (**B**) in human breast adenocarcinoma cells of MCF-7 line under doxorubicin treatment. 1 – control; 2 – doxorubicin, 1 µM; 3 – doxorubicin, 1 µM+Na_2_SeO_3_, 0.1 µM; 4 – doxorubicin, 1 µM+selenomethionine, 0.1 µM; 5 – doxorubicin, 1 µM+D-pantethine, 25 µM.

Next, we attempted to confirm the results obtained at the molecular level by studying the production of the superoxide radicals in human breast adenocarcinoma cells of MCF-7 line under the action of doxorubicin and the antioxidants (Figure 5A). Doxorubicin (1 µM) caused a 10-fold increase in the production of superoxide anion compared to control (untreated cells), whereas addition of sodium selenite (0.1 µM) reduced ROS production to nearly baseline level. Selenomethionine (0.1 µM) and D-pantethine (25 µM) showed weaker but also pronounced inhibitory effect on ROS levels in MCF-7 cells (Figure 5A). Therefore, all applied antioxidants acted by inhibiting the production of superoxide anion in target tumor cells.

It is known that ROS are involved in the induction and regulation of apoptosis signaling pathways in various (including tumor) cells (21,22). To ensure that antioxidants’ inhibition of production of superoxide anions induced by doxorubicin can also lead to further suppression of apoptosis, cytomorphological study of human breast adenocarcinoma cells of MCF-7 line under the action of doxorubicin in combination with the antioxidants was performed (Figure 5B). Doxorubicin (1 µM) caused a development of typical apoptosis hallmarks – hypercondensation of nuclear chromatin and cell contraction, whereas sodium selenite almost completely restored the cytomorphological phenotype of MCF-7 cells to normal. Selenomethionine and D-pantethine demonstrated similar effect (Figure 5B). Thus, inhibition of doxorubicin-induced production of toxic superoxide anions under action of applied antioxidants also stopped the switching of apoptosis by this drug.

### D-pantethine and selenomethionine inhibit oxidative stress induced in rat erythrocytes by doxorubicin action *in vivo*

Finally, we tried to confirm our conclusions from the treatment of tumor cells *in vitro* in an animal study on adult male Wistar rats. A redox-status of experimental animals and redox efficiency of applied compounds were evaluated by biochemical assays for analysis of glutathione system in rat erythrocytes. Nile blue sorption and methylene blue adsorption were applied for estimation of plasma membrane damage of erythrocytes under the doxorubicin action. Doxorubicin treatment led to a decrease in the redox-potential of the erythrocytes, and it also sharply decreased GSH/GSSG ratio, while selenomethionine and D-pantethine rapidly restored both parameters almost to the control level (Table 1). Doxorubicin caused a small damage to plasma membranes of erythrocytes, as detected by Nile blue sorption (Table 2), but this damage was partially restored under the action of selenomethionine and D-pantethine (Table 2). Doxorubicin also induced oxidative stress (measured by an increase in the level of diphenyl-aminoreaсting substances), which was eliminated by a simultaneous application of D-pantethine or selenomethionine (Table 3). Total (effective) redox potential in the erythrocytes was rapidly decreased under doxorubicin action, while selenomethionine totally blocked such action (Table 1). Surprisingly, a combined action of doxorubicin with D-pantethine led to a sharp decrease in the amount of protein sulfhydryl groups in blood plasma of treated rats (Table 3).

**Table 1:** Glutathione level and its redox potential in erythrocytes of Wistar rats with combined administration of doxorubicin (Dx), D-pantethine, and selenomethionine (SeMet) (mean ± standard deviation, n = 8)

Parameter	Control	Dx	D-panthetine +Dx	SeMet+Dx
Reduced glutathione, µM/gHb	4.55 ± 0.40	4.33 ± 0.40	4.95 ± 0.43^†^	4.84 ± 0.50^†^
Oxidized glutathione, µM/gHb	0.057 ± 0.006	0.091 ± 0.004*	0.073 ± 0.009*	0.073 ± 0.007*
Redox ratio of reduced and oxidized glutathione	80.3 ± 5.8	51.0 ± 4.8*	67.7 ± 3.9*†	68.08 ± 4.62*^†^
Overall glutathione, µM/gHb	4.66 ± 0.41	4.51 ± 0.39	5.09 ± 0.44*	4.98 ± 0.28
Redox potential, mV	-327.5 ± 3.3	-320.7 ± 3.5*	-326.4 ± 3.0†	-325.9 ± 3.1^†^

*P < 0.05 relative to the control.

†*P* < 0.05 relative to Dx.

**Table 2:** Indicators of Nile blue (NB) and methylene blue (MB) dye sorption in erythrocytes of Wistar rats with combined administration of doxorubicin (Dx), D-pantethine, and selenomethionine (SeMet) (mean ± standard deviation, n = 8)

Parameter	Control	Dx	D-panthetine +Dx	SeMet+Dx
Sorption coefficient (NB), conventional units (c.u.)	39.37 ± 3.32	43.86 ± 3.66*	42.62 ± 4.42	38.53 ± 5.43
Sorption coefficient (MB), c.u.	8.80 ± 0.69	9.87 ± 0.95	9.28 ± 0.99	9.21 ± 1.17
Sorption coefficient (NB), c.u./gHb	132.1 ± 8.9	141.2 ± 17.0	132.8 ± 17.5	127.7 ± 13.4
Sorption coefficient (MB), c.u./gHb	29.6 ± 3.1	31.8 ± 4.5	28.8 ± 3.3	30.7 ± 4.0
Absorption ratio (NB), **%**	84.8 ± 1.1	86.2 ± 1.0*	85.8 ± 1.4	84.4 ± 1.9
Absorption ratio (MB), %	74.5 ± 1.5	76.6 ± 1.8	75.4 ± 2.0	75.2 ± 2.3

*P < 0.05 relative to the control.

**Table 3:** Changes in oxidative stress indices in the blood plasma of Wistar rats under combined administration of doxorubicin (Dx), D-pantethine, and selenomethionine (SeMet) (mean ± standard deviation, n = 8)

	N,N-Dimethyl-p-phenylenediamine	Protein sulfhydryl-groups	
	conventional units /mL of blood plasma	µM/mL of blood plasma	µM/mg of protein
Control	397.5 ± 13.1	131.4 ± 9.5	1.66 ± 0.10
Dx 5 mg/kg	455.3 ± 27.6*	129.0 ± 9.1	1.68 ± 0.16
D-pantethine +Dx	393.4 ± 24.1^†^	105.2 ± 7.9*^†^	1.21 ± 0.11*^†^
SeMet+Dx	332.9 ± 26.9^†^	121.9 ± 5.9	1.49 ± 0.16*

*P < 0.05 relative to the control.

†*P* < 0.05 relative to Dx.

### Multidirectional effects of D-pantethine and selenomethionine toward succinate dehydrogenase activity and coenzyme A content in the rat liver under doxorubicin action

**Figure 6:**
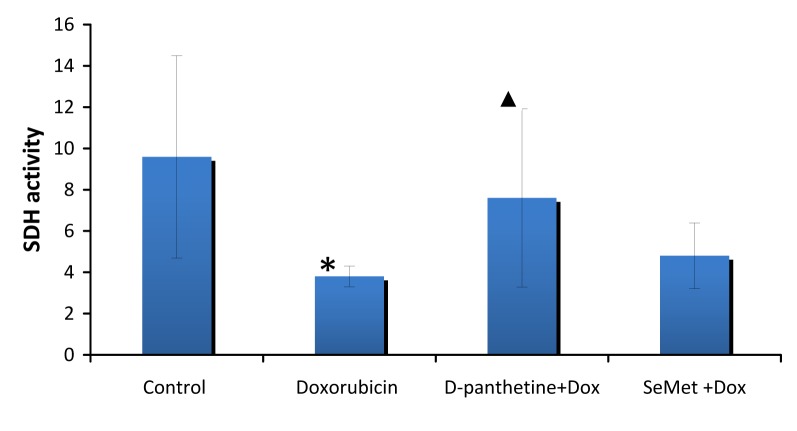
D-pantethine, but not selenomethionine, restores the activity of succinate dehydrogenase (SDH) (nmol/mg protein* min) in the rat liver under doxorubicin treatment. *P < 0.05 relative to the control, triangle represents *P* < 0.05 relative to doxorubicin.

In order to verify the involvement of mitochondrial oxidation processes in realization of toxic effect of doxorubicin, succinate dehydrogenase (SDH) activity in the liver of experimental animals was studied (Figure 6). A 2.5-fold decrease in the activity of this enzyme was detected under the action of doxorubicin, while D-pantethine restored it to control level. In contrast, there was no significant effect of selenomethionine on the SDH activity (Figure 6).

**Figure 7:**
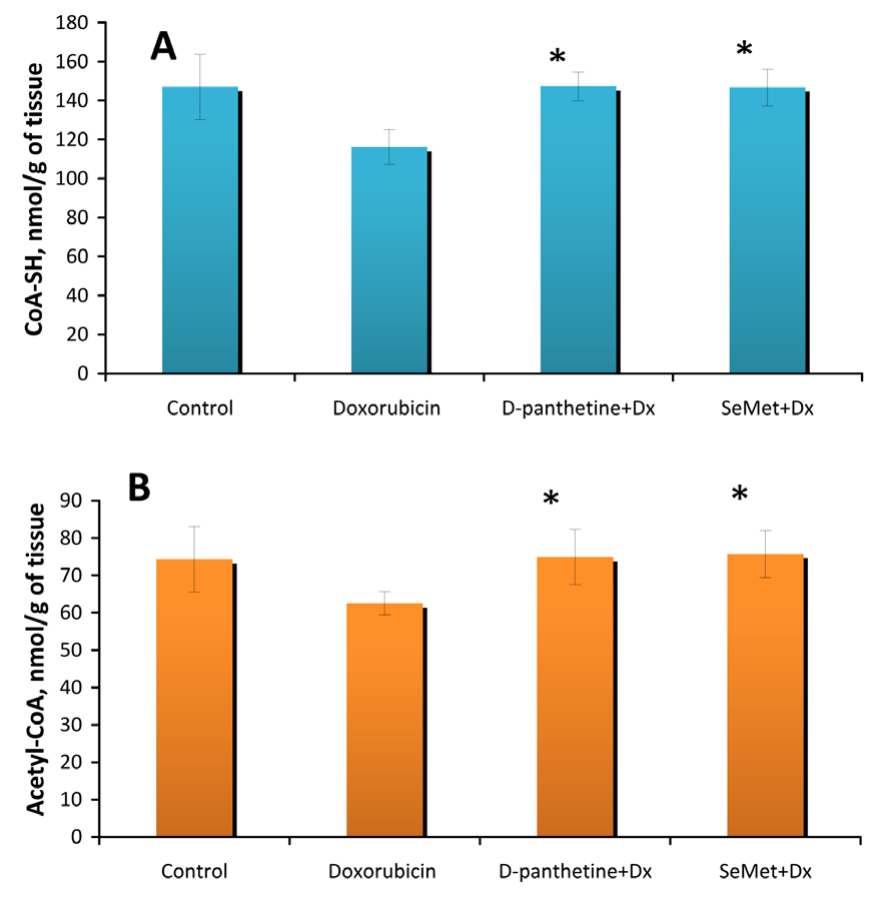
Selenomethionine and D-pantethine restore content of coenzyme A-SH (**A**) and acetyl-coenzyme A (**B**) in the liver of normal rats after administration of doxorubicin. *P < 0.05 relative to doxorubicin.

The content and ratio of CoA fractions are considered to be essential indicators of the enzymatic systems involved in energy metabolism and detoxification processes. Doxorubicin treatment led to a pronounced and significant drop in the fraction of free CoA, as well as of acid-soluble CoA in the rat liver tissue (Table 4). D-pantethine (CoA precursor) did not show any protective effect, whereas selenomethionine showed a trend toward normalization of the studied parameters (Table 4). Since these data were obtained by using cyclic phosphotransacetylase assay, they cannot exclude a direct effect of doxorubicin in that analytical system. To avoid this problem, HPLC study of the content of free form of CoA (CoA-SH) and a short-chain acyl-CoA derivative (acetyl-CoA) in the rat liver was carried out. The obtained results confirmed the CoA-mediated mechanism of cytotoxic action of doxorubicin and demonstrated highly protective effect of the D-pantethine and selenomethionine, which restored a quantity of both CoA-SH and acetyl-CoA to the control level (Figure 7).

**Table 4:** Effect of intragastric administration of D-pantethine and selenomethionine (SeMet) on the contents of coenzyme A fractions (nmol/g of tissue) in the liver of rats under doxorubicin (Dx) treatment (CoA – coenzyme A) (mean ± standard deviation, n = 8)

	Acid-soluble CоА	Short-chain acyls of CоА	Free CоА
Control	292 ± 13	140 ± 9	152 ± 8
Doxorubicin	246 ± 12*	129 ± 7	117 ± 5*
D-pantethine+Dx	258 ± 14	136 ± 8	122 ± 6*
SeMet+Dx	262 ± 14	131 ± 7	131 ± 7

*P < 0.05 relative to the control.

## Discussion

Cardio- and nephrotoxicity of doxorubicin significantly limit its application in cancer chemotherapy. These negative side effects are caused by an excessive production of superoxide anions in the mitochondria of cells treated with doxorubicin (4). While the antitumor activity of this antibiotic is mainly implemented via inhibition of the DNA topoisomerase II (3), the induction of ROS is only a supplementary, and, at the same time, harmful mechanism accompanying doxorubicin action toward target cells, particularly those which contain a large number of mitochondria – cardiomyocytes and hepatocytes. This is why a search for new approaches capable of reducing the effects of the oxidative stress caused by the doxorubicin is essential for clinical medicine.

It is known that ROS are also involved in the mechanisms of anticancer activity of cisplatin, however, their impact is much weaker compared to the effect of doxorubicin (1). p53/ROS/p38α MAPK cascade was shown to be essential for cisplatin-induced cell death in HCT-116 cells (23). However, in our experiments antioxidants caused only a statistically insignificant decrease (7%) of cisplatin-induced anticancer action toward this cell line. We found that ROS scavengers did not affect the consequences of cisplatin treatment. Thus, their application together with cisplatin in chemotherapy regimens does not seem to be effective. In contrast, sodium selenite, selenomethionine, and D-pantethine significantly modulated cytotoxic activity of doxorubicin, thus, we studied doxorubicin action in more detail.

Although sodium selenite, selenomethionine, and D-pantethine possessed similar ROS scavenging activity both *in vitro* and *in vivo*, the molecular mechanisms of their action was different. We showed that all applied antioxidants modulated the effect of doxorubicin toward human colon carcinoma cells of HCT-116 line and human T-leukemia cells of Jurkat line via inhibiting cytotoxic activity of this drug by 15%-30%. It should be noted that a slight increase in sodium selenite and selenomethionine concentration from 0.1 µM to 0.25 µM (both were not toxic to target cells) significantly suppressed their cytoprotective effects, while higher doses of these compounds (5 µM, still low toxicity toward tumor cells *in vitro*) had an opposite effect, notably increasing doxorubicin toxicity (data not shown). Similar results – cytoprotective activity at low doses and enhancement of cytotoxic action at higher doses – were also observed for D-pantethine effect. To summarize, these results suggest an existence of different mechanisms of action of the studied antioxidants toward tumor cells depending on the concentration of these agents.

After finding that selenium-containing compounds partially decreased cytotoxic action of doxorubicin, but not that of cisplatin, toward tumor cell lines with different drug sensitivity, it was reasonable to study in more detail antioxidant modulation of the sensitivity of tumor cells with the MDR phenotype to doxorubicin and cisplatin.

Resistance acquisition of tumor cells to anticancer drugs is a serious problem in clinical practice. It was found that during one year of chemotherapy, resistance to anticancer drugs develops in 30%-50% of cancer patients (24). Drug resistance is a multi-factorial and complex phenomenon (25), which results in a significant decrease in drug accumulation in cells by limiting their uptake, enhancing efﬂux, or affecting membrane lipids such as ceramide (26). These changes lead to: 1) inhibition of the programmed cell death (apoptosis) that is induced by most anticancer drugs (27); 2) activation of the mechanisms of general response that detoxify drugs and repair DNA damage (28); 3) alterations in cell cycle and its checkpoints that render cancer cells relatively resistant to cytotoxic effects of drugs. High expression of P-glycoprotein, MRP-1 protein, and bcrp in human tumors is considered to be the first sign of negative prognosis for cancer patients (10). Another mechanism of development of drug resistance is related to genetic defects in the structure of *Bax* and *p53* genes, whose products play an important role in the regulation of cell cycle and apoptosis (29). Tumor cells with defects in these genes exhibit higher invasive potential, increased ability to metastasize, and are more resistant to chemotherapy (30).

Combined action of anticancer drug with sodium selenite, selenomethionine, and D-pantethine showed a cytoprotective activity toward tumor cell lines that are sensitive to the action of doxorubicin and a reverse effect (enhancing of cytotoxic activity of the doxorubicin) toward drug-resistant tumor cells with various defects (overexpression of P-glycoprotein or p53 and Bax knockouts). The dual activity of these antioxidants can be very important in clinical practice, since using these compounds as part of cancer chemotherapy regimens could reduce negative side effects of anticancer drugs toward normal cells and strengthen drug action in drug-resistant tumor cells.

Finally, we wanted to confirm our findings regarding cytoprotective effect of the selected antioxidants toward cultured mammalian cells treated with anticancer drugs in the *in vivo* experiments on rats treated with highly toxic doxorubicin combined with the physiological doses of selenomethionine and D-pantethine. In our studies, antioxidants and doxorubicin doses were selected on the basis of literature data (1). 1000 μg dose of L-selenomethionine/kg bw/d (equivalent to 400 μg selenium/kg bw/d) was reported to be a no-observed-adverse-level (NOAEL) in a 13-week study in rats (1). At the same time, no genotoxicity and no carcinogenicity has been observed for D-pantethine action, and no developmental toxicity in mice and rats at up to 600 mg/kg bw/d doses (31). 16 mg/kg of i.p. injected doxorubicin was its LD_50_ dose in rats (*http://www.lclabs.com/MSDS/D-4000MSDS.php4*). In the *in vivo* experiments, a cumulative dose of 1 mg/kg selenomethionine (5 daily gavages of 200 µg/kg), 2000 mg/kg dose of D-pantethine (5 daily gavages of 400 mg/kg), and 5 mg/kg dose of doxorubicin injected i.p. were used. 1:5 ratio for selenomethionine-doxorubicin and 1:400 ratio for doxorubicin-D-pantethine were applied. One can see that the used selenomethionine and D-pantethine doses are lower than the corresponding NOAEL doses, while the doxorubicin dose was 3 times lower than its LD_50_ in rats. Thus, no adverse effects of selenomethionine and D-pantethine alone were expected.

0.05-0.25 µM range of selenomethionine dose (depending on cell line sensitivity), 1-50 µM range of D-pantethine, and 0.5-5 µM dose of doxorubicin were applied in the *in vitro* experiments. Average ratio was 1:5 for selenomethionine:doxorubicin and 1:100 for doxorubicin:D-pantethine. Thus, the same ratio of selenomethionine and doxorubicin doses was applied in both *in vitro* and *in vivo* experiments in order to ensure the replicability of the obtained results. A 400:1 ratio of D-pantethine (2000 mg/kg) to doxorubicin (5 mg/kg) in the *in vivo* experiments was 4 times higher compared to the 100:1 ratio *in vitro* (50 µM of D-pantethine to 0.5 µM doxorubicin) due to non-toxicity of D-pantethine in mice.

The results of our *in vivo* studies in rats confirmed the results of *in vitro* studies on ROS-scavenging activity of sodium selenite and D-pantethine. Both these compounds blocked the induction of the oxidative stress *in vivo*. This was revealed by testing the restoration of redox-potential of erythrocytes and oxidative status of human plasma, which were increased under doxorubicin action. Besides, CoA level was restored in the liver of the experimental animals treated with doxorubicin. Selenomethionine did not have a statistically significant effect on the activity of SDH involved in Complex II of the mitochondrial respiratory chain of the liver, while D-pantethine strongly impacted the activity of that enzyme. This suggests various molecular mechanisms underlying cytoprotective activity of selenium derivatives and D-pantethine.

In conclusion, the obtained results suggest a high potential of using selenomethionine and D-pantethine in tumor chemotherapy regimens in combination with a very toxic drug – doxorubicin. A protective effect of the antioxidant agents during cytotoxic action of doxorubicin was demonstrated *in vitro* (cultured tumor cells) and *in vivo* (laboratory rats). In case of cisplatin, such effect was not pronounced. These results might be important for planning further pre-clinical trials of the combinatory treatment schemes using anticancer drugs and specific antioxidant compounds.
